# Vaccination in twin pregnancies: comparison between immunization before conception and during pregnancy

**DOI:** 10.1038/s41598-024-61504-6

**Published:** 2024-05-11

**Authors:** Ran Svirsky, Moran Landau Rabbi, Ramzia Abu Hamad, Adi Sharabi-Nov, Nadav Kugler, Narina Galoyan, Nataly Zilberman Sharon, Hamutal Meiri, Ron Maymon, Osnat Levtzion-Korach

**Affiliations:** 1 Department of Obstetrics and Gynecology, Shamir (Assaf Harofeh) Medical Center, Zerifin, Israel; 2 Medical Genetic Unit, Department of Obstetrics and Gynecology, Samson Assuta Ashdod University Hospital, Ashdod, Israel; 3 Faculty of Health Sciences, Ben-Gurion University of the Negev, Be’er Sheva, Israel; 4 Clinical Chemistry Lab, Shamir Medical Center, Zrifin, Israel; 5 Department of Statistics, Ziv Medical Center, and Tel Hai Academic College, Safed and Tel Hai, Israel; 6 PreTwin Screen Consortium and TeleMarpe Ltd, Tel Aviv, Israel; 7 School of Medicine, Faculty of Medicine and Health Science, Tel Aviv University, Tel Aviv, Israel; 8 Hospital Management, Shamir (Assaf Harofeh) Medical Center, Zerifin, Israel

**Keywords:** BNT162b2 Pfizer/BioNTech vaccination, COV-2-Trimeric S-IgG -b1, Neutralizing antibodies, PCR, SARS-CoV-2, Twin pregnancy, Immunology, Diseases, Health care, Medical research

## Abstract

To evaluate the development of neutralizing Anti-Spike Protein IgG (Anti-S-IgG) during twin pregnancies before conception vs. during pregnancy. In this prospective study, three blood samples were collected from pregnant women and subjected to anti-S-IgG immunodiagnostics. The patient’s medical records, including vaccination and PCR test results, were collected from the hospital’s electronic database. Age-matched non-pregnant women were used as a control group. We enrolled 83 women with twin pregnancies. 49 women were vaccinated before conception, 21 women were vaccinated during pregnancy, and 13 were not vaccinated. Of the 13 women who weren’t vaccinated, three became positive during pregnancy, and all three were severely ill. By contrast, in women who were vaccinated during or before pregnancy, COVID-19 infection during pregnancy caused only mild symptoms. A ten-fold lower level of neutralizing Anti-S-IgG in the 3rd trimester was observed in healthy women who were vaccinated before conception and remained healthy until discharge from the hospital after delivery 1605 (IQR: 763–2410) compared to the healthy women who were vaccinated during pregnancy 152 AU/mL (IQR: 54–360). This difference was higher among women who were infected by COVID-19 (as verified by a positive PCR test). The third-trimester level of neutralizing Ant-S-IgG in the infected group was 4770 AU/mL (4760–6100) in infected women vaccinated before conception compared to those vaccinated during pregnancy who had 70 AU/mL (IQR: 20–170) (*p* < 0.001). In women vaccinated at 13–16 weeks gestation, neutralizing Anti-S-IgG at 20–22 weeks went up to 372 AU/mL (IQR: 120–1598) but rapidly dropped to 112 AU/mL (IQR: 54–357) at 28–30 weeks, (*p* < 0.001), a faster decline than in women vaccinated at a median 22 weeks before conception. Being infected by COVID-19 before conception was linked to having low Anti-S-IgG levels during pregnancy, whereas being infected by COVID-19 during pregnancy led to a very high response in the 3rd trimester. In twin pregnancies, significantly lower neutralizing Anti-S-IgG levels were observed in women vaccinated during pregnancy compared to those vaccinated before conception, whether infected or not infected by COVID-19. A full course of vaccination before conception is recommended.

Trial registration. ClinicalTrials.gov Protocol Registration and Results System (PRS) Receipt Release Date: October 4, 2021. https://clinicaltrials.gov/ ID: NCT04595214.

SARS-CoV-2 virus infection is associated with severe acute respiratory syndrome, and it remains a major global concern^1^. Shortly after its initial outbreak, studies indicated that pregnant women infected with SARS-CoV-2 (“Covid-19”) developed more severe symptoms than age-matched non-pregnant women^2–4^. Its severity in pregnancy was attributed to having higher body mass compared to age-matched non-pregnant women and constricted chest space due to the growing uterus, which are all considered to exacerbate the load on the lungs and place an elevated burden on the cardiac output^5^. It has been argued that since pregnant women experience increased immune tolerance to avoid fetal rejection, this process should also increase their vulnerability to severe symptoms^6^. The immune system's overreaction to the viral infection (“the cytokine storm”) may thus exacerbate the known increase of cytokines already reported for other pregnancy-related complications such as preeclampsia, early IUGR, and gestational diabetes^5,6^. Previous studies have indicated a 3-to-tenfold prevalence of severe COVID-19 during pregnancy^7^.

In Israel, the authors of this study were among the first in the world to formulate guidelines for vaccinating all pregnant women during gestation, including those carrying singletons and twins. These guidelines were published soon after the vaccine became available in the country. Several reports showed a high rate of vaccination among pregnant women compared to other countries^8^. Extensive proactive efforts by medical staff to explain the safety of the vaccine to their patients and evidence of infant protection by vaccination were major drivers for vaccine acceptance^9^. However, no study has examined the development of neutralizing IgG following BNT-162b2 Pfizer/BioNTech vaccination or SARS-CoV-2 virus infection in twin pregnancies. This study examined the longitudinal development of neutralizing antibodies after vaccinations and viral infection before conception and during twin pregnancy to fill this gap.

## Methods

### Sample

Pregnant women aged 18 and over carrying two live fetuses were enrolled after providing their written informed consent. Women were excluded if they previously had twins that vanished or triplets that were spontaneously reduced to twins. The gestational week was determined by the last menstrual period and by the crown-rump length^10^. Women who subsequently lost one fetus or underwent twin reduction to singleton due to fetal defects remained in the study.

Medical, demographic, and pregnancy history were collected at enrolment, and each clinical visit included a complete anatomical evaluation by sonography, measurements of blood pressure, and three blood drawings in the 1st, 2nd, and 3rd trimesters. Delivery and pregnancy outcomes were retrieved from the hospital medical records of the delivery and the neonatal clinics. and, from interviews with patients if they delivered in other hospitals.

### Serological testing

Blood was collected from all women for serological testing at gestational weeks 11–13, 20–22, and 28–30 to test for anti-spike (S) antibodies (Anti-S-IgG) to SARS-CoV-2 infection. The test was performed using the LIAISON® SARS-CoV-2 (DiaSorin, Saluggia, Italy) with 97.4% and 98.5% sensitivity and specificity, respectively. Samples were considered negative for antibody titers at < 13 AU/mL^11^.

A real-time Polymerase Chain Reaction (rtPCR) test was performed in the case of suspected infection either locally or in public clinics using the FDA-approved TaqPath Combo Kit targeting the N2, ORF1Ab, and S genes^12^. Infection was then confirmed by a quantitative test of Anti-N-IgG to the SARS-CoV-2 nucleoprotein using Elecsys® immunoassay (Roche Diagnostics, Mannheim, Germany), according to the manufacturer’s instructions. All methods were performed in accordance to the relevant guidelines and regulations. The sensitivity and specificity of this assay are both > 99%. The cutoff index (COI) was defined as < 1.0 for non-reactive samples^13^. Serological values are expressed as arbitrary units per milliliter (AU//mL according to the standardization of the World Health Organization (WHO)^14^.

### Vaccination

Pregnant women vaccination with BNT162b2 Pfizer/BioNTech vaccine for SARS-CoV-2 beginning in February 2021, and women at gestation age (GA) 15 and 19 got the first two doses. Eventually, more and more women conceived after being vaccinated before pregnancy as once installed, vaccination was free and offered to all, with wide access everywhere; all unvaccinated women in our study actively refused vaccination. The median vaccination before pregnancy was 22 weeks. Vaccination records were retrieved from the patient’s vaccination cards and verified against the national registry records of the government. The time after immunization was calculated from the end of the second vaccination. We also collected data on the third booster shot but not all women took it. The delay between vaccination and testing was recorded..

Anti-S-IgG level of age-matched non-pregnant women of the hospital employee were retrieved from the hospital records. These women were tested immediately before, one month after, and three months after their third vaccination by BNT162b2 Pfizer/BioNTech.

Data on singleton vaccination was extracted from publications^15,16^.

### Statistical analysis

The SPSS statistical package version 29 (IBM) was used to conduct Kruskal–Wallis and Mann–Whitney non-parametric tests to compare the groups for the continuous variables. Continuous variables were not normally distributed hence we provide medians (Me) and interquartile ranges (IQR). Chi-square tests were applied to categorical values presented as n (%). A *p*-value < 0.05 was considered statistically significant.

### Ethics approval

This study is a part of the Erapermed project on the evolution of twin pregnancies (JTC2019-61)^17^. Date was extracted from one of the study sites at Shamir Medical Center. An amended ethics approval was specifically obtained by “Shamir local ethic committee” to evaluate the serological response to BNT162b2 Pfizer/BioNTech vaccination to SARS-CoV-2 (Trial # 0043–20-ASF, Israel Ministry of Health Authorization # 202,016,632). All participants expressed their consent to participate in the study by signing a specialized add-on consent form approved by the ethical committee, and all methods were performed in accordance with the relevant guidelines and regulations of serological and PCR testing as issued by Israel Ministry of Health.

## Results

### Cohort characteristics

**Figure 1:**
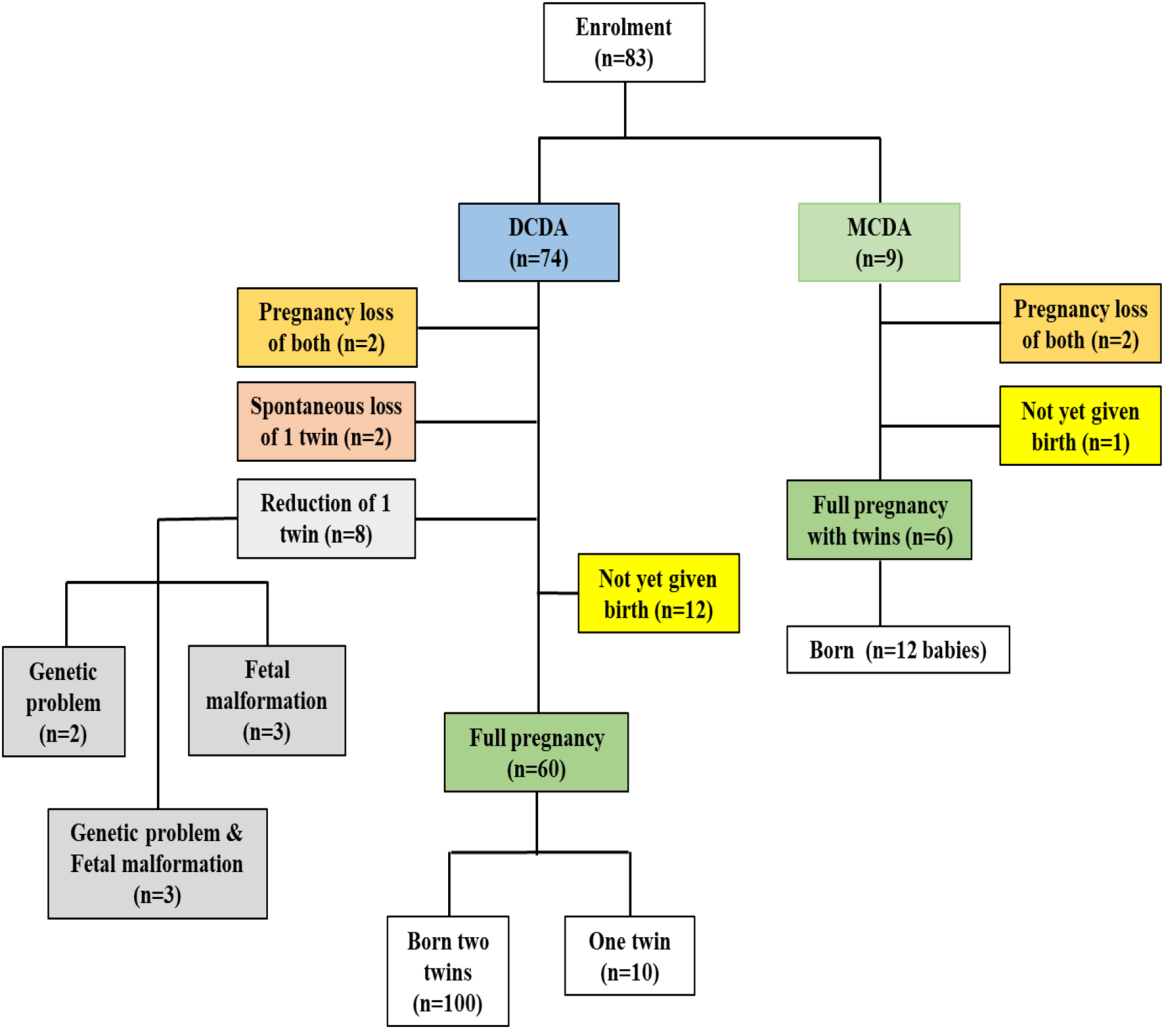
Flow chart of enrolment to delivery.

**Figure 2:**
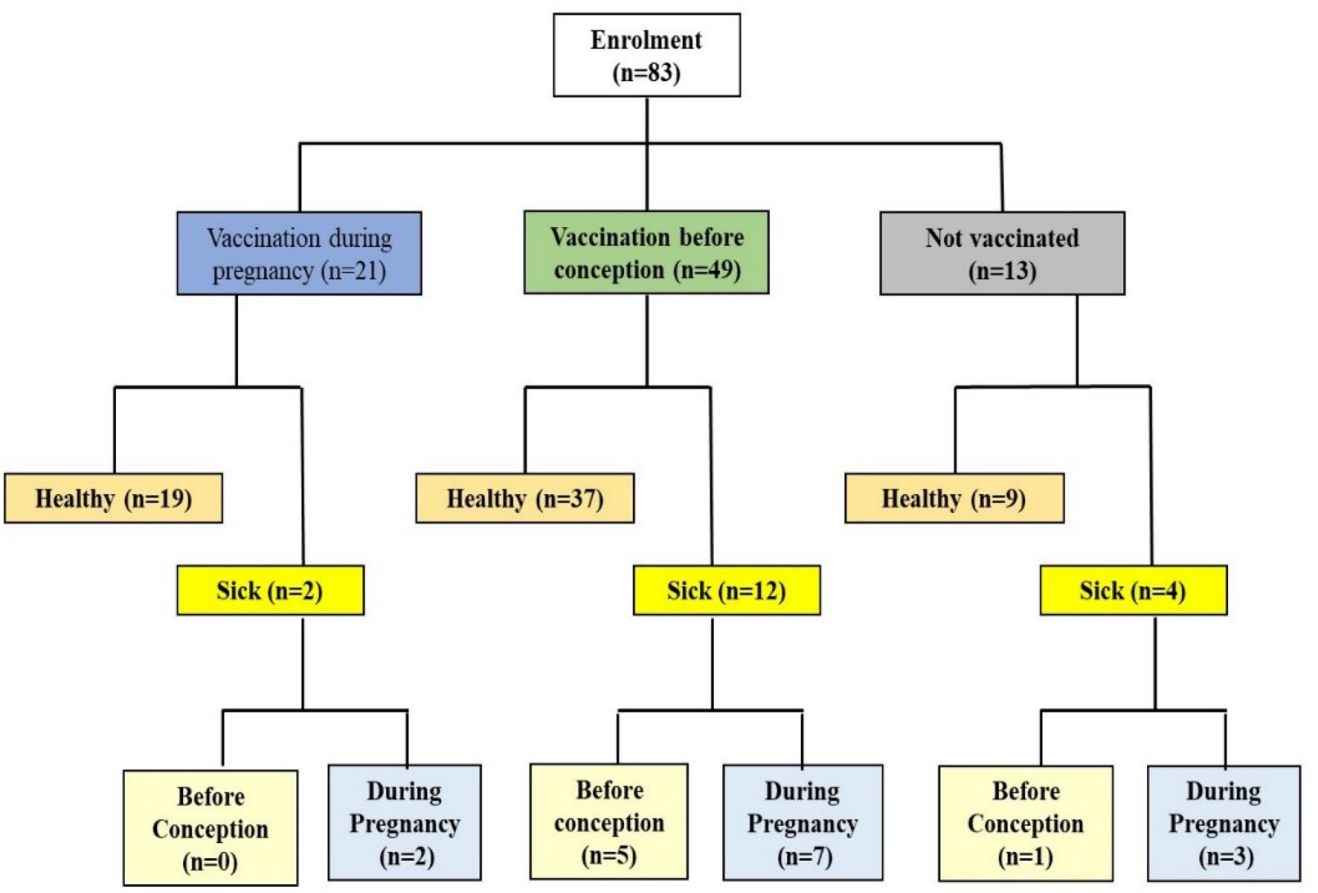
Flow chart for vaccination and sickness.

A total of 83 women with twin pregnancies were enrolled from December 2020 to March 2022. Of these, 74 were carrying di-chorionic di-amniotic (DCDA), and 9 had mono-chorionic di-amniotic (MCDA) twins (Fig. 1). Of these women, 49 women were vaccinated before conception, 21 were immunized during pregnancy, and 13 were non-vaccinated (Fig. 2).

The median maternal age at enrolment was 34.3 years, the median GA at enrollment was 12.3 weeks, and the median BMI was 24.9 (all values were centered on the medians). Conception was spontaneous for all MCDC and 47.3% of the DCDA (p < 0.001). There were 37.2% nulliparous, and the proportion between Jews and Arabs was as it is in the regional population (Top of Table 1). Chronic medical complications (diabetes mellitus, hyper-or-hypothyroidism, etc.) were rare, and none had chronic hypertension or cardiovascular diseases. All these features correspond to the general characteristics of our Twin Clinic population^17,18^.

**Table 1:** Maternal characteristics at enrollment and at delivery.

Characteristic	All (n = 83)	DCDA (n = 74)	MCDA (n = 9)	*p*
Enrollment				
MA, years (Me, IQR)	34.3 (30.4–37.3)	34.7 (30.6–37.3)	31.5 (25.1–35.2)	0.137
GA at enrollment, wks. (Me, IQR)	12.3 (11.7–12.9)	12.1 (11.7–12.9)	12.8 (12.3–13.0)	0.231
BMI, kg/m^2^ (Me, IQR)	24.9 (22.0–29.7)	24.9 (22.0–29.7)	23.4 (21.1–27.0)	0.383
Ethnicity, n (%)				
Jews	78 (94.0)	70 (94.6)	8 (88.9)	0.573
Arabs	5 (6.0)	4 (5.4)	1 (11.1)	
Diabetes mellitus, n (%)	2 (2.4)	2 (2.7)	0 (0)	0.596
Asthma, n (%)	6 (7.2)	5 (6.8)	1 (11.1)	0.718
Hypothyroidism, n (%)	3 (3.6)	3 (4.1)	0 (0)	0.514
Hyperthyroidism, n (%)	3 (3.6)	3 (4.1)	0 (0)	0.514
Cardiovascular diseases, n (%)	0 (0)	0 (0)	0 (0)	-
Other complications, n (%)	12 (14.5)	10 (13.5)	2 (22.2)	0.633
Conception method, n (%)				
Spontaneous	38 (46.9)	29 (39.1)	9 (100)	0.011
IVF other ART	32 (39.5)	32 (45.1)	0 (0)	
Ovulation induction	4 (4.9)	5 (5.6)	0 (0)	
Sperm donation	7 (8.6)	7 (9.9)	0 (0)	
Nulliparous, n (%)	30 (36.1)	26 (35.1)	4 (44.4)	0.787

Pregnancy complications	(n = 66)	(n = 60)	(n = 6)	*p*
GDM, n (%)	14 (21.2)	12 (20.0)	2 (33.3)	0.936
PE, n (%)	5 (7.5)	5 (8.3)	0 (0)	0.602
Preterm delivery, < 37 wks., n (%)	36 (54.5)	31 (53.3)	5 (83.3)	0.723
Delivery by Cesarean section, n (%)	36 (54.5)	32 (58.2)	4 (50.0)	0.865

Live newborn	n = 122	n = 110	n = 12	*p*
GA at live delivery, wks. (Me, IQR)	36.4 (34.9–37.3)	36.7 (35.0–37.3)	34.6 (32.4–36.6)	0.110
Birthweight, gr. (Me, IQR)	2400 (2275–2530)	2400 (2260–2500)	2295 (1670–2630)	0.301
1 min APGAR < 7, n (%)	7 (7.1)	7 (7.9)	0 (0)	0.610
5 min APGAR < 7, n (%)	0 (0)	0 (0)	0 (0)	–
Newborn gender, female, n (%)	58 (47,5)	48 (43.6)	10 (83.33)	0.266
Newborn weight < 2500, n (%)	69 (55.6)	60 (54.5)	9 (75.0)	0.812
Newborn weight < 1500, n (%)	8 (6.6)	4 (3.6)	4 (33.3)	0.012

Newborn complications after delivery	n = 122	n = 110	n = 12	*p*
Any complications^#^, n (%)	19 (15.6)	16 (14.5)	3 (25.0)	0.714
NICU days (Me, IQR)	14 (7–19)	11 (5–19)	16 (–)	–
Loss after delivery, n (%)	0 (0)	0 (0)	0 (0)	–

All values are presented as n (%) or Median (interquartile range (IQR)). # Respiratory support.

Values of all di-chorionic di-amniotic (DCDA) twins and mono-chorionic, mono-amniotic (MCDA) twins were compared using Kruskal–Wallis non-parametric test or Chi-square test.

BMI—body mass Index, IVF- in-vitro fertilization, ART- assisted reproduction technology, APGAR—standardized score to evaluate infants shortly after birth as developed by Virginia APGAR and include in five criteria: activity (tone), pulse, grimace, appearance, and respiration. For each criterion, newborns can receive a score from 0 to 2, and integrated a score of 10. NICU—Intensive care units, GDM- gestational diabetes melilotus, PE- preeclampsia.

### Pregnancy outcomes

All MCDC pregnancies were delivered by Caesarean section (CS) compared to 57.1% of the DCDA. The remainder of the DCDA twins were delivered vaginally (40.5%) or via tool-assisted delivery (Middle of Table 1).

Among the DCDA pregnancies, 50 women delivered twins (100 babies) and ten delivered singletons. This was due to spontaneous demise (2 cases), and selective reduction due to major genetic or structural malformation (8 cases (Fig. 1). Also, there were 2 cases of complete pregnancy loss, and 12 women didn’t deliver at the time we concluded the study. (Fig. 1, left side).

Among MCDA twins, 6 women delivered 12 babies, two lost their pregnancy spontaneously, and 1 didn’t deliver at the time of study conclusions. (Fig. 1, right side).

Altogether 122 babies were born. (Fig. 1). There were 47.5% females (71.4% in the MCDC). No significant differences were found between the birthweight of babies born in MCDA and DCDA pregnancies. Of the live newborns, there were 55.6% who had low birthweight (< 2,500 g), but only 6.6% had very low birth weight (< 1500 g). The majority of the newborns had a normal APGAR score at 1 and 5 min, the duration of NICU days was 10 days, and no newborn death after delivery (Bottom of Table 1).

### Pregnancy complications

The rate of gestational diabetes melilotus (GDM) was 21.2%, preeclampsia was 5%, and preterm delivery (delivery < 37 weeks) was 55.4%, (Middle of Table 1) all of which are within the range of known values for twins in Israel^17,18^.

### Vaccination and susceptibility to COVID-19

The study included 83 patients, of which 70 were vaccinated, and 13 were not (Table 2). Of the non-vaccinated women, nine were healthy (69.2%), and four became PCR positive (30.7%). One was diagnosed before conception, and three (23.1%) contracted COVID-19 during pregnancy (Table 2). All three were admitted to the hospital in the third trimester with very severe symptoms. Luckily, they delivered healthy babies.

**Table 2:** Maternal COVID-19 illness, vaccination and serology.

Characteristic		All (n = 83)	DCDA (n = 74)	MCDA (n = 9)	*p*
COVID-19, n (%)					
Healthy (PCR^-^)		65 (79.3)	57 (77.9)	8 (88.9)	0.592
Sick (PCR^+^)		18 (21.6)	17 (22.2%)	4 (11.1)	
PCR^+^ before conception		8 (9.6)	5 (6.75)	3 (33.3)	
PCR^+^ during pregnancy		13 (15.9)	12 (16.7)	1 (10.0)	
Wks. of PCR^+^ before conception, wks. (Median, IQR)		35.0 (24–(41))	35.0 (24–(41))	–	–
GA at PCR + during pregnancy, wks. (Median wks, IQR)		24.0 (17.0–27.0)	24.1 (16.0–27.5)	24.0 (–)	–
Vaccinations, n (%)					
Unvaccinated		13 (15.7)	9 (11.4)	4 (12.5)	
Healthy		9 (69.2)	5 (55.6)	4 (100)	
Contracted COVID-19		4 (30.8)	4 (69.2)	0 (0)	
Before conception		1 ( 7.8)	1 (11.1)	0 (0)	
During pregnancy		3 (23.1)	3 (33.3)	0 (0)	
Vaccinated					
All vaccinated		70 (84.3)	65 (87.8)	5 (55.5)	
Before conception		49 (70.0)	44 (67.7)	5 (100.0)	
During pregnancy		21 (30.0)	21 (32.3)	0 (0.0)	
Vaccinated healthy					
Total healthy		56 (80.0)	52 (80)	4 (80.0)	
Vaccinated before conception		37 (66.1)	33 (63.5)	4 (100.0)	
Vaccinated during pregnancy		19 (33.9)	19 (36.5)	0 (0.0)	
Vaccinated COVID-19^+^		14 (16.9)	13 (17.6)	1 (11.1)	
Vaccinated PCR +					
Before	Before	5 (35.7)	5 (38.5)	0 (0.0)	
Before	During	7 (50.0)	6 (46.2)	1 (100.0)	
During	During	2 (14.3)	2 (15.4)	0 (0.0)	
During	Before	0 (0.0)	0 (0.0)	0 (0.0)	

All values are presented as n (%) or median and interquartile range (IQR).

As the first and second vaccinations during pregnancy were at GA 15 and 19 weeks, Testing was performed at GA 12.1 (pre-vaccination), 22.4 (3 weeks after the second vaccination), and at 29 weeks (10 weeks after the second vaccination). Pre-pregnancy vaccination was performed at a median of 22.7 weeks pre-conception. Thus, testing at the 1st, 2nd, and 3rd trimesters performed at 34.8, 45.1, and 51 weeks after the second pre-conception vaccination.

According to Table 2, among the 70 women who were vaccinated, 56 (80%) were healthy. Of the 14 patients who were immunized but viral infected (PCR +), there were five were vaccinated before pregnancy and PCR + before conception (5%), seven were vaccinated before conception and PCR + during pregnancy (10%), and two were vaccinated during pregnancy and the viral infected (2.9%) (Table 2). There was no case of PCR + before pregnancy who was PCR positive during pregnancy. Altogether, vaccination appears to protect women who were pregnant with twins; if they were PCR + , their symptoms were mild and need no special admission to the hospital before delivery.

## Serological response

### Response as a function of time of vaccination

#### Time of vaccination

**Figure 3:**
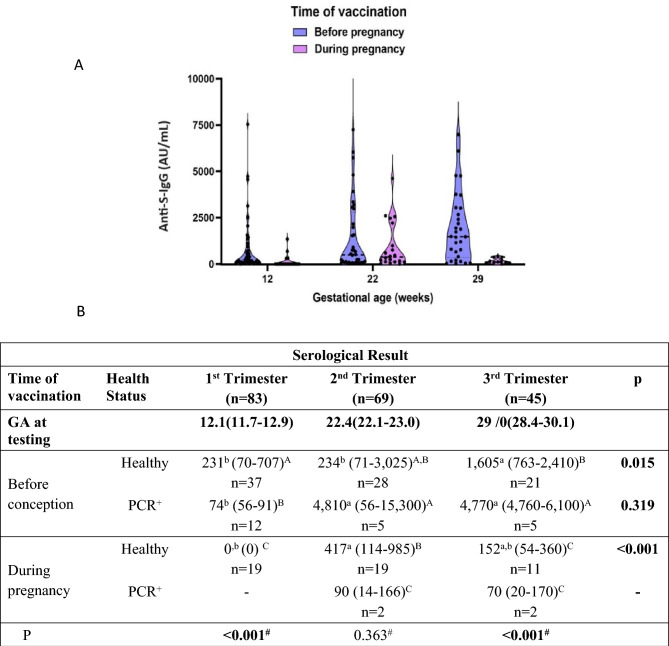
The level of neutralizing Anti-S-IgG (AU/mL) in the 3 trimesters as a function of the time of vaccination, (**A)** Violin plot of the serological differences between patient vaccinated before conception and during pregnancy in the 3 trimesters. First and second vaccinations during pregnancy were performed at a median gestational weeks (GA) 16 and 19. Testing was performed at GA 12.1weeks (pre-vaccination), 22.4 (3 weeks after the 2nd vaccination), and at 29 weeks, 10 weeks after the second vaccination. The median preconception vaccination week was 22.7. Thus, testing at the 1st, 2nd and 3rd trimesters, respectively, should be performed 34.8, 45.1, and 51 weeks after the second pre-conception vaccination. (**B)** Median neutralizing Anti-S-IgG (AU/mL) with inter quartile range (IQR). Statistical significance between trimesters is shown to the right according to the Kruskal–Wallis non-parametric test. a-b: different letters represent statistical significance of individual groups where a is largest, b, is lower and ab is in between a and b. The P at the bottom is the statistical significance between the groups in each trimester, and the letters to the right of the IQR are A—the largest, B—smaller, A,B—in between A and B, and C—the smallest.

In women who were vaccinated before conception (violin plot, Fig. 3A), the level of neutralizing Anti-S-IgG doubled from the first to the second trimester (*p* = 0.05), and tripled from the second to the third trimester (*p* < 0.01), reaching a median of 1475 AU/mL (IQR: 392–3020), reflecting a sevenfold increase during pregnancy (overall, *p* = 0.012). Nevo L et al. demonstrated that vaccination with BNT-162b2 Pfizer/BioNTech during singleton pregnancies was followed by a third-trimester Anti-S-IgG level of 798 AU/mL (IQR: 424–1623)^19^, indicating that vaccination during pregnancy with twins compared to singleton pregnancy is generating a weaker level of Anti-S-IgG, which also decay faster.

There were 56 women who were vaccinated and healthy—37 were vaccinated before conception and 19 during pregnancy (Table 2). Among healthy vaccinated before conception (median—22 weeks before conception), the neutralizing Anti-S-IgG significantly increases from 231 AU/mL (IQR: 70–707) in the 1st trimester to 1605 AU/mL (IQR: 763–2410) in the third trimester (*p* < 0.015). Among women vaccinated during pregnancy (gestational week 15–19), the levels increased from 0 in the 1st trimester to 417 AU/mL (IQR: 114–985) in the 2nd trimester (3–4 weeks after vaccination), and it then decreases back to 152 AU/mL (IQR: 54–360) (Fig. 3B). This decay of neutralizing Anti-S-IgG reflects the immunosuppression state of the pregnant women.

The main result of our study was the X10 and above levels of Anti-S-IgG when vaccination was before conception compared to vaccination during pregnancy, as manifested in the 3rd trimester (Fig. 3A,B). The serological response in women vaccinated during pregnancy showed an increase from 0 in the first trimester to a median of 372 AU/mL in the second trimester, which rapidly dropped to one-third of this amount in the third trimester to a median of 112 AU/mL (IQR: 54–357) (Fig. 3A,B). In third-trimester singleton pregnancies vaccinated during pregnancy, the Anti-S-IgG level is 380 AU/mL (IQR: 65.35–1442.5), which is higher compared to twins (*p* < 0.005)^14,15^. It appears that during pregnancy, the immune system is suppressed, and this immunotolerance is higher in twins.

No significant differences were found between DCDA and MCDA twins for any of the tested parameters (Table 2, supplementary Fig. 1).

#### Viral infection and time of vaccination

**Figure 4:**
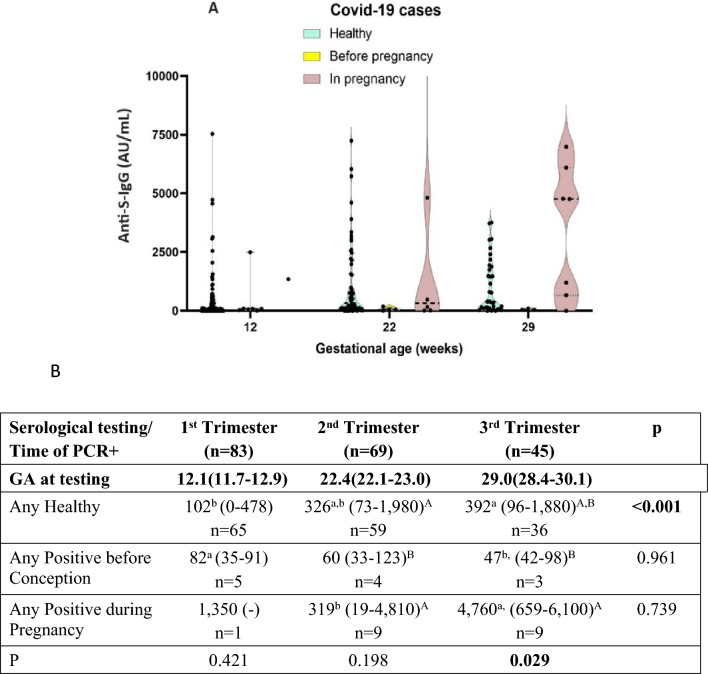
The level of neutralizing Anti-S IgG (AU/mL) in the PCR-positive cases in the 3 trimesters as a function of the time of vaccination (**A)** Violin plot of Median levels of neutralizing Anti-S-IgG (AU/mL). The green group are patients who were PCR negative to COVID-19 (healthy) whether immunized or not. PCR + patients are divided according to the time of PCR positive test, whether before conception or during pregnancy. (**B)** Medians with interquartile range (IQR) of the above groups. The statistical significance (P) to the right of the table compares values across trimesters according to the Kruskal–Wallis non-parametric test, and the letters to the left of the values are: a—the larger, b—the smaller, a,b – in between. The P value at the bottom is comparing values at the same trimester. The letters to the right of the IQR are—A—the largest, B—smaller, and A,B—in between.

While 14 vaccinated women (out of 70—20% compared to 31% among non-vaccinated),all had very mild disease. There were 5 who were vaccinated and infected before conception, and all had low level of neutralizing Anti-S-IgG throughout pregnancy. The 9 who got infected during pregnancy had a very high level of Anti-S-IgG (Fig. 4A,B). iInfection during pregnancy is accompanied by very high level of neutralizing Anyti-S-IgG to protect these women and their babies.

### Serological response in non-pregnant women

**Figure 5:**
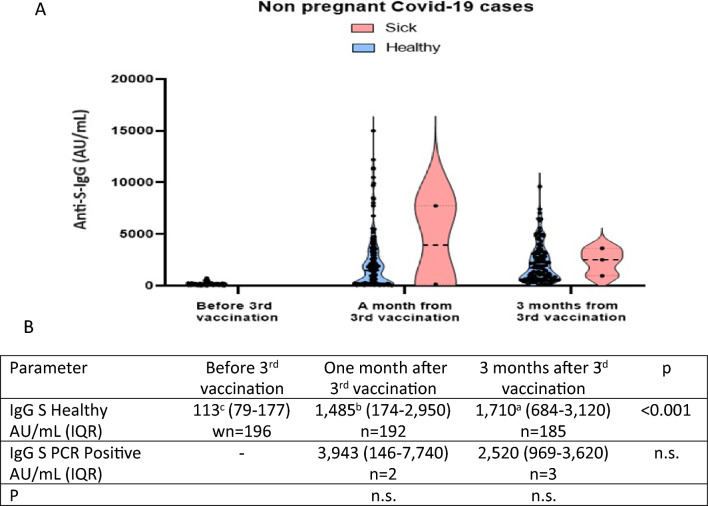
The level of neutralizing Anti-S-IgG (AU/mL) in non-pregnant women as a function of time from the third immunization (**A)** Violin Plot among non-pregnant women who tested for neutralizing Anti-S- IgG (AU/mL) before and one and three months after the third immunization. Values are plotted for the PCR positive and negative subjects. (**B)** The median and interquartile range (IQR) of the non-pregnant women. All women were between 25 and 42 years old. The statistical significance (P) to the right represents the differences according to the Kruskal–Wallis non-parametric test for the difference between the time points across the group at 1 and 3 months after the third vaccination. Letters represent statistical significance where a is the a—largest, b—the lower, and c—the lowest.

We compared our twin pregnant women neutralizing Anti-S-IgG levels to the results of the sample of non-pregnant women of a matched tested in the same period of time. The non-pregnant women were tested one and three months after the third vaccination. The one-month delay (vaccination to serological testing) among non-pregnant women was similar to the 3–4 weeks delay between vaccination and second-trimester testing of twin pregnancy immunized during pregnancy. Anti-S-IgG is 1,485 AU/mL (IQR: 174–2950) among the non-pregnant, compared to 417 AU/mL (IQR: 114–985), *p* < 0.03) in pregnant women, emphasizing the weakness of the immune protection during pregnancy (Figs. 3 and 5). The three-month delay is close to the delay between pregnancy vaccination and third-trimester testing (10–12 weeks). Here, the differences are even greater among the healthy nonpregnant women; Anti-S-IgG levels were 1,710 vs. 152 in the pregnant women who were vaccinated during pregnancy, showing the immunosuppressive state response of pregnancy (Figs. 3 and 5). The story for those vaccinated before conception is totally different. Although tested at 45.1- and 51-week delay between vaccination and testing, the third-trimester level of Anti-S-IgG is indistinguishable from the level of non-pregnant tested after three months (1605 vs. 1710).

## Discussion

We see now that COVID-19 is turning into a seasonal viral infection and multiple variants as predicted by the World Health Organization (WHO)^1,20^, and at seasonal waves new cases are added every day. Hence, information related to the anti-viral vaccination and the viral infection—remains crucial. Studies have shown that maternal vaccination effectively protects pregnant women, their fetuses, and newborns from becoming infected^8,11^. Extensive studies support the importance of vaccination of gravid subjects before and during pregnancy and have confirmed its efficacy in reducing the rate of infection and protecting them from severe symptoms^21^. However, no studies have explored twin pregnancies^22^. The current study provides the first detailed evidence on the natural history of neutralizing Anti-S-IgG in twin pregnancies.

A main result of our study is that in twin pregnancies vaccinated before conception the levels of neutralizing Anti-S-IgG in the 3rd trimester is 10 × higher than in those vaccinated during pregnancy. Vaccination before conception is accompanied by a faster increase, and a slower decay of neutralizing Anti-S-IgG compared to vaccination during pregnancy. These results underscore the importance of immunization before conception. This is important since other studies have shown that the strength of the response of the neutralizing Anti-S-IgG may influence the transfer of neutralizing Anti-S-IgG to the newborn^15,16^,

Pregnant women with twins have a larger placental mass that further reduces the chest cavity, creating a greater pressure on the lungs, and on the cardiovascular system. This is accompanied by higher incidence of preeclampsia, IUGR, gestational diabetes. The latter appears to be related to immunotolerance and here we see how the immune suppression impair an effective Anti-S-IgG protection^5,6,23,24^.

One explanation for the lower relative increase and the rapid decay of Anti-S-IgG in twin gravid women vaccinated during pregnancy is the potential uptake by the larger placental mass^25^. Placental uptake of Anti-S-IgG may also explain the similar Anti-S-IgG levels found in DCDA and MCDA. It remains to be seen why this was apparently not a factor in cases where vaccination took place before conception^26,27,28^.

These findings also reinforce the importance of vaccination, given that the women who were immunized before conception and during pregnancy developed very mild symptoms, whereas the unvaccinated women who contracted COVID-19 had severe inflammatory and respiratory symptoms that required hospitalization. Overall, these results confirm that in twins, as in singleton pregnancies, being immunized protects pregnant women from severe cases of COVID-19.

## Limitations

The study was conducted as the COVID-19 pandemic developed. Thus, our quasi-naturalistic study constraints led to variations in the time of vaccinations and contributed considerably to the individual variance in the levels of neutralizing Anti-S-IgG. Diversity was also contributed by the wave of the pandemic, which influenced patient behavior. Due to the study's timing, we could evaluate cases who were vaccinated during and before pregnancy. Twins are the minority of pregnancies (3% of the population) , and very large cohorts were hard to ascertain since the rate of both spontaneous and IVF pregnancies went down during the pandemic. However, this actually enabled us to review multiple scenarios. Since COVID-19 is still present, we feel that the clinical question regarding the time of vaccination in pregnancy, especially twin pregnancy, is important, and our study answers this important question.

Another limitation is that data regarding a cell-mediated immune response that would have enhanced our understanding of the mother's immune response and immunity was not collected. Further studies are thus warranted.

## Conclusion

Immunizing pregnant women is crucial to protect mothers and their newborns from severe disease during the COVID-19 pandemic. Our study provides evidence that as in singletons, gravid women with twins benefitted from being vaccinated, and if they were contracted with the virus, they only experienced mild symptoms. Given the high risk of COVID-19 during pregnancy, especially in multiple pregnancies, the findings show that vaccination is important for pregnant women, and is ten times more effective if taken prior to the conception, since the immune system of pregnant women is suppressed. Further studies with larger cohorts are warranted.

### Supplementary Information

Supplementary Information.

## Data Availability

The datasets generated during and/or analyzed during the current study are available from the corresponding author upon reasonable request.

## Abbreviations

BNT: 162B2 Pfizer/BioNTech vaccination—the messenger RNA vaccination against COVID-19 that was developed by the companies Pfizer and BioNTech
IgG: Immuno-globulin type G
Anti-S-IgG: Immunoglobulin type G (IgG) against the spike protein of Covid-19 virus
Anti-N-IgG: Immuno-globulins targeting the nucleuse N2 protein of Covid-19
Anti-N-IgG SARS-COV-2: The seventh known coronavirus to infect people, also known as COVID-19
COV-2-Trimeric S-IgG -b1: A test developed by DISORIN company named the LIAISON® SARS-CoV-2 Trimeric S IgG assay that enables the reliable detection of IgG to Trimeric spike (S) protein IgG—the body’s natural defense response against SARS-CoV-2-Trimeric S-IgG -b1
MCDA twins: Mono-chorionic diamniotic twin pregnancies
DCDA twins: Di-chorionic diamniotic twin pregnancies
PCR: Polynuclear chain reaction
rtPCR: Real-time PCR
IUGR: Intra Uterine Growth Restriction
GDM: Gestational diabetes melilotus
FDA: Food and Drug Administration
IQR: Interquartile range
WHO: World Health Organization
BMI: Body Mass Index
ART: Assisted Reproduction Reaction
APGAR: Standardized score to evaluate infants shortly after birth as developed by Virginia APGAR and includes five criteria: Appearance, Pulse, Grimace, Activity, and Respiration. For each criterion, newborns can receive a score from 0 to 2, and an integrated a score could be 10 max
NICU: Newborn intensive care unit
GA: Gestational age in weeks
MA: Maternal age
