# Supplementary material for: Vaccination in twin pregnancies: comparison between immunization before conception and during pregnancy
Source: Sci Rep. 2024 May 11;14:10813. doi: 10.1038/s41598-024-61504-6 (PMC11088702; doi:10.1038/s41598-024-61504-6)
Supplement: Supplementary file 1 — Supplementary Information. [file 41598_2024_61504_MOESM1_ESM.docx]

**Supplementary Table 1: Maternal serology of Anti-S-IgG (AU/mL)**

|  | **All**  **(n=83)** | **DCDA**  **(n=74)** | **MCDA**  **(n=9)** | p |
| --- | --- | --- | --- | --- |
| **1^st^ trimester** |  |  |  |  |
| GA at testing, wks. (IQR) | 12.1 (11.7-12.9) | 12.1 (11.7-12.7) | 13.0 (11.7-13.3) | 0.147 |
| Anti-S-IgG AU/mL (IQR) |  |  |  |  |
| Healthy | 99 (0-477) | 85 (0-560) | 104 (45-119) | 0.720 |
| PCR+ Before conception | 74 (31-1291) | 74 (31-1291) | - | - |
| PCR+ During pregnancy | 716 (82-1350) | 716 (82-1350) | - | - |
| **2^nd^ Trimester** |  |  |  |  |
| GA at testing, wks. (IQR) | 22.4 (22.1-23.0) | 22.4 (22.1-23.0) | 22.7 (21.4-23.3) | 0.867 |
| Anti-S-IgG AU/mL (IQR) |  |  |  |  |
| Healthy | 283 (71-1775) | 372 (91-1980) | 61 (19-1530) | 0.249 |
| PCR+ Before conception | 56 (10-183) | 56 (10-183) | - | - |
| PCR+ During pregnancy | 472 (166-4810) | 472 (166-4810) | - | - |
| **3^rd^ Trimester** |  |  |  |  |
| GA at testing, wks. (IQR) | 29.0 (28.4-30.1) | 29.0 (28.4-30.1) | 28.9 (28.1-29.9) | 0.695 |
| Anti-S-IgG AU/mL (IQR) |  |  |  |  |
| Healthy | 395 (104-1,810) | 397 (139-1,740) | 30 (7-3,760) | 0.492 |
| PCR+ Before conception | 70 (42-98) | 70 (42-98) | - | - |
| PCR+ during pregnancy | 4,765 (659-6,100) | 4,770 (659-6,100) | 4,760 (-) | - |

All values are presented as median (interquartile range (IQR)) and are compared between Mono and Di-chorionic twins (MCDA and DCDA). To the right is the P value according to Kruskal Walis, non-parametric analysis. In each trimester the values of PCR positive during pregnancy are higher
